# A Natural Gas Energy Metering Method Based on Density-Sound Velocity Correlation

**DOI:** 10.3390/s26010343

**Published:** 2026-01-05

**Authors:** Bin Zhang, Zhenwei Huang, Wenlin Wang, Junxian Wang, Dailiang Xie, Ying Cheng, Yi Yang

**Affiliations:** 1 College of Metrology Measurement and Instrument, China Jiliang University, Hangzhou 310018, China; p23020854125@cjlu.edu.cn (B.Z.); dlxie@cjlu.edu.cn (D.X.); p23020854004@cjlu.edu.cn (Y.C.); p24020854140@cjlu.edu.cn (Y.Y.); 2 Flow and Energy Metrology & Testing Institute, Energy Environment and Life Health Metrology Center, Zhejiang Institute of Quality Sciences, Hangzhou 310018, China; wangwenlin111@163.com; 3 College of Metrology & Measurement Engineering, China Jiliang University College of Modern Science and Technology, Yiwu 322002, China; wangjunxian@cjlu.edu.cn

**Keywords:** natural gas, energy metering, calorific value, physical property correlation, compression factor, machine learning, model switching, real-flow validation

## Abstract

This paper proposes a method for metering natural gas energy based on the correlation between density and sound velocity. The technique integrates physical property correlation models with measured parameters, such as temperature, pressure, sound velocity, and density, to accurately predict the compression factor and the ideal volume calorific value of natural gas under operating conditions. The volume flow is corrected using the compression factor, which enables precise metering of natural gas energy through the adjusted volume flow and calorific value. To develop a high-precision physical property correlation model, a natural gas dataset comprising 10,000 sample sets is first constructed for model training and testing. Multiple machine learning algorithms are then employed to build predictive models. Analysis of the experimental results led to the development of a model-switching strategy based on the ranges of input features, which substantially enhanced prediction accuracy. For the compression factor model, the mean absolute error (MAE), root mean square error (RMSE), mean absolute percentage error (MAPE), and coefficient of determination (R^2^) were 0.00118, 0.0030, 0.14%, and 0.9987, respectively. The corresponding indicators for the calorific value model were 0.1583, 0.331, 0.44%, and 0.9736. The proposed method is finally validated using a natural gas real-flow test bench. The results demonstrated maximum prediction errors of 0.061% and 1.19% for the compression factor and calorific value, respectively, while the maximum relative energy error across four gas samples was 1.21%. These results indicate that the method can effectively achieve accurate natural gas energy metering in practical operating conditions.

## 1. Introduction

Energy metering, which accounts for both the quality and quantity of natural gas, has gradually become the mainstream method for settlement in natural gas trading [1,2]. Accurate measurement of natural gas volume and calorific value is critical to ensuring the reliability of energy metering [3,4]. However, factors such as temperature, pressure, composition, and the proportion of each component in natural gas can influence its volume and calorific value, making high-precision energy metering a significant challenge. Currently, energy metering predominantly relies on gas component analysis methods [5]. These methods are used on gas chromatograph analyzers as the core equipment, which are associated with high costs, long measurement cycles, and operational complexity [6]. Consequently, gas chromatograph-based energy metering is difficult to implement on a large scale, and its accuracy can be compromised in gas sources with substantial component fluctuations.

Related studies have highlighted the advantages of using physical property correlation technology to determine energy metering parameters, including lower costs, higher speeds, and minimal environmental requirements [7,8]. This approach utilizes the inherent relationships between the physical properties of natural gas and the parameters needed for energy metering. By measuring key physical properties, it is possible to predict related parameters and thus achieve energy metering [9]. For instance, the Southwest Research Institute (SWRI) proposed a correlation-based method to directly determine the calorific value of natural gas using sound velocity, pressure, temperature, and inert gas content (Nitrogen(N_2_), Carbon dioxide(CO_2_)) as input parameters for ultrasonic flow meters [10]. Additionally, optical methods for determining calorific value have also received significant attention. Kojima et al. [11] proposed a method correlating natural gas sound velocity with the visible light spectrum to determine calorific value; preliminary verification by RIKEN Corporation in Japan re-ported relative deviations of –0.45% to 0.38% compared to theoretical values. In a similar manner, Kamimoto et al. [12] applied Raman spectroscopy and tunable diode laser absorption spectroscopy (TDLAS) to explore the relationships between spectral absorption and calorific value for mixed gases. Ying et al. [13] developed a Raman analysis approach, incorporating a spectral automatic decomposition algorithm and a quantitative analysis model, further improving the accuracy of natural gas calorific value determination. Overall, physical property correlation technology has become a valuable tool in natural gas energy metering, effectively reducing dependence on traditional gas chromatographs. Research and applications to date have primarily focused on calorific value determination. However, in practical pipeline transportation, natural gas often exists under medium- to high-pressure conditions, where it experiences a certain degree of compression. Consequently, accurate determination of the compression factor is equally critical for volumetric metering. According to relevant standards [14], traditional compression factor calculations require detailed information about gas composition. Physical property correlation technology, which often lacks detailed composition data, faces limitations in its application for energy metering under these conditions.

Intelligent learning algorithms, with their capabilities for non-linear modeling, automatic extraction of high-dimensional features, and data-driven adaptive learning, overcome the limitations of traditional methods in handling complex non-linear and multi-variable problems. As a result, they have become essential tools for addressing real-world challenges [15,16,17]. In energy metering, these algorithms are essential for constructing predictive models related to physical property correlation technology, thus advancing the application and development of this technology. Karpash et al. [18] proposed a neural network-based method to predict the calorific value of natural gas, using nitrogen concentration, carbon dioxide concentration, and sound velocity as input parameters to estimate calorific values under standard temperature and pressure conditions. By training the network on a database of 95 natural gas mixture samples, the model achieved a relative error of only 2.4% compared to standard calorific values. Chamkalani et al. [19] developed a CSA-LSSVM model for predicting the compression factor of both sulfur-containing natural gas and conventional natural gas, utilizing a dataset of 4756 natural gas samples. Rigorous statistical validation confirmed the model’s reliability, providing a robust solution for compression factor prediction. Farzaneh-Gord et al. [20] trained an artificial neural network with 30,000 randomly composed natural gas samples, using pressure, temperature, and the Joule-Thomson (JT) coefficient as inputs to predict the compression factor. Finally, validation with regional natural gas data against the GERG-2008 results demonstrated an overall average absolute percentage deviation (AAPD) of less than 0.7%. In their study, Shaghayegh et al. [21] combined a genetic algorithm (GA) with temperature, pressure, nitrogen, and carbon dioxide concentrations to predict natural gas components and thermodynamic properties. The predicted values deviated by less than 0.8% from the standard AGA8Eos calculations. Farzaneh Gord et al. [22] further optimized the traditional three-input neural network by incorporating calorific value alongside temperature, pressure, and density to predict the natural gas compression factor. The enhanced model achieved a mean absolute percentage error (MAPE) of 0.2%, a root mean square error (RMSE) of 0.0028, and a coefficient of determination (R^2^) of 0.9943, demonstrating the effectiveness and reliability of this optimization strategy. Hu et al. [23] addressed the issue of quality fluctuations in complex natural gas pipelines by establishing a deep learning model for the prediction of dynamic calorific value. This model efficiently extracted influential factors and provided rapid predictions from real-time data, achieving a calorific value prediction accuracy that exceeds 99% while requiring only 1% of the processing time of conventional physical models. Dong et al. [24] proposed a multilayer perceptron neural network (MLPNN) approach using temperature, pressure, and sound velocity measured by an ultrasonic flow meter for energy metering. Based on 1003 real data points, the MLPNN model achieved maximum relative errors of −0.44%, 1.90%, and 2.39% for the compression factor, calorific value, and daily cumulative energy, respectively.

In summary, intelligent learning algorithms provide significant advantages in predicting the thermal and physical properties of natural gas, providing strong support for advancing the application of physical property correlation technology in energy metering. Nevertheless, current research exhibits several limitations. Many proposed prediction models rely on the concentrations of non-calorific components, such as carbon dioxide and nitrogen, as input parameters. Detecting these components in practical energy metering scenarios is often difficult and costly. Second, most studies have focused on predicting individual thermal property parameters, with relatively limited systematic research on natural gas across the entire energy metering process. Third, the majority of models are trained using high-quality natural gas samples. When faced with significant variations in natural gas composition or elevated levels of non-calorific gases, predictive accuracy and generalization notably decrease, restricting limiting the models’ ability to adapt to dynamic changes in thermal properties under complex operating conditions.

This paper proposes a natural gas energy metering method based on the correlation between density and sound velocity to address these challenges. The technique highlights physical parameters that are easy to measure while effectively characterizing the compression factor and calorific value of natural gas across various operating conditions. This approach aims to reduce metering errors arising from the ‘one-to-many’ phenomenon in physical properties and energy metering parameters. A theoretical database is constructed using on standard methods, providing data support for training intelligent learning models. By evaluating and comparing the predictive performance of multiple algorithms, the most effective model is identified and optimized, and its accuracy is subsequently validated through real-flow natural gas experiments. This method allows for real-time, convenient, and reliable metering of natural gas energy, providing a practical and strong pathway for the broader application of physical property correlation technology in energy metering.

## 2. Materials and Methods

**Figure 1:**
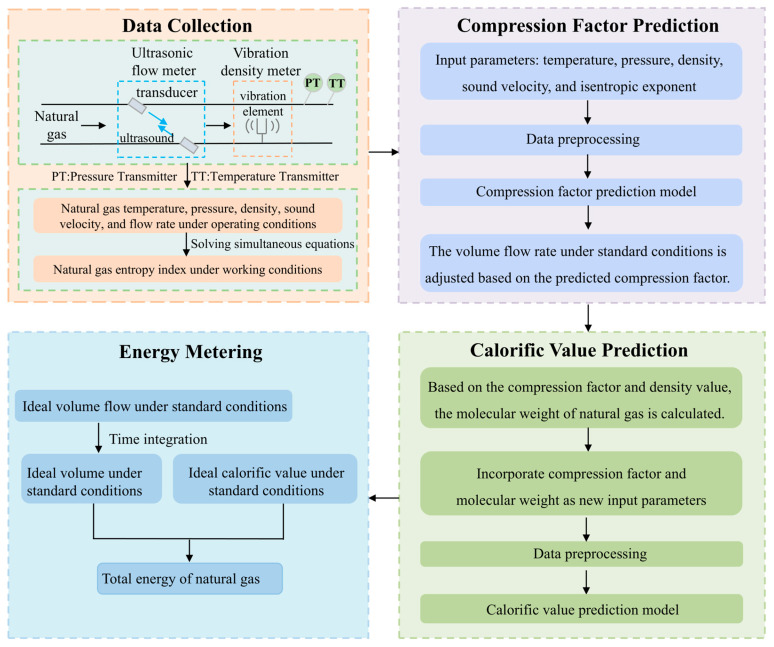
Flowchart of the natural gas energy metering method based on the density-sound velocity correlation approach.

Figure 1 illustrates the implementation process of a natural gas energy metering method based on the correlation between density and sound velocity. First, temperature and pressure sensors, ultrasonic flow meters, and vibrating density meters are installed in the gas pipeline to acquire real-time measurements of temperature, pressure, sound velocity, and density. These measurements are then used to calculate the isentropic index. These physical parameters are preprocessed and input into the compression factor prediction model to determine the compression factor, which, combined with the density measurement, is used to calculate the molecular weight of the gas. Finally, the pre-processed temperature, pressure, sound velocity, density, isentropic index, compression factor, and molecular weight are input into the calorific value prediction model to estimate the standard ideal volume calorific value. Multiplying this value by the standard volume, obtained from the corrected standard flow rate integral, yields the total natural gas energy and completes the metering process.

### 2.1. Feature Correlation Analysis

#### 2.1.1. Analysis of Key Parameters for Energy Metering

Accurate energy metering requires precise determination of both the gas volume and the calorific value per unit volume (hereinafter referred to as ‘calorific value’). The product of these two quantities represents the energy released from the complete combustion of natural gas. The principle is expressed in Equation (1):

(1) $$E=\int_{t_{0}}^{t_{n}}e_{s}(t)dt=\int_{t_{0}}^{t_{n}}q_{s}(t)\cdot H_{s}(t)dt,$$

where E is the total energy of the natural gas (MJ); $t_{0}$ and $t_{n}$ are the initial and final moments, respectively (s); $e_{s}(t)$ is the instantaneous energy flow as a function of time (MJ/s); $q_{s}(t)$ is the volume flow rate as a function of time (m^3^); and $H_{s}(t)$ is the calorific value as a function of time (MJ/m^3^); The subscript s denotes standard reference conditions (hereinafter referred to as ‘standard conditions’), considering a temperature of 293.15 K and a pressure of 101.325 kPa.

Since actual pipeline conditions (“operating conditions”) differ from standard conditions, the volume flow rate must be corrected accordingly. Furthermore, the compressibility of natural gas causes deviations between the volume of real gas and the volume of ideal gas (*Z* = *V*(actual)/*V*(ideal)) [25]. Therefore, a compression factor, along with gas state parameters under operating conditions, is introduced to correct the flow value, as illustrated in Equation (2):

(2) $$q_{s}=f\cdot q_{o}=\frac{P_{o}\cdot T_{s}}{T_{o}\cdot P_{s}}\cdot \frac{Z_{s}}{Z_{o}}\cdot q_{o},$$

where q denotes the volume flow rate, m^3^/h; f is the dimensionless volume flow correction parameter; P is the pressure, MPa; T is the thermodynamic temperature, K; and Z is the dimensionless compression factor. The subscript o indicates the operating conditions.

Natural gas is primarily a methane-dominated mixture. Standards specify the ideal calorific value of each component under standard conditions [26]. The overall calorific value is calculated using Equation (3):

(3) $$H_{s}=H_{s}^{o}/Z_{s}=[\sum_{i=1}^{n}\left(H_{s,i}^{o}\cdot x_{i})\right]/Z_{s},$$

where $H_{s}^{o}$ is the ideal calorific value under standard conditions, MJ/m^3^; $H_{s}$ is the calorific value under standard conditions, MJ/m^3^; $H_{s,i}^{o}$ is the ideal calorific value of each component in the natural gas under standard conditions, MJ/m^3^; $x_{i}$ is the molar fraction of each component, %mol/mol; and n is the total number of components in natural gas.

Combining Equations (1)–(3) yields the total energy expression, as shown in Equation (4):

(4) $$E=\int_{t_{0}}^{t_{n}}e_{s}(t)dt=\int_{t_{0}}^{t_{n}}q_{s}\cdot H_{s}dt=\int_{t_{0}}^{t_{n}}\frac{P_{o}\cdot T_{s}}{T_{o}\cdot P_{s}}\cdot \frac{H_{s}^{o}}{Z_{o}}\cdot q_{o}dt,$$

Equation (4) highlights that eliminating reliance on gas chromatography for energy metering requires accurate prediction of the compression factor under operating conditions and the calorific value under standard conditions. In this study, measurable natural gas properties that can characterize both factors are selected as input parameters for the intelligent learning algorithm, enabling precise prediction and complete energy metering.

#### 2.1.2. Compression Factor Feature Correlation Analysis

This study selects the temperature, pressure, density, and sound velocity of natural gas under operating conditions as key measurable physical properties. These properties are not only straightforward to measure but also allow for high-frequency data acquisition. Temperature and pressure can be monitored in real time using dedicated sensors, while density and sound velocity can be measured online via a vibrating gas density meter and an ultrasonic flow meter, respectively [27,28,29]. These two quantities are expressed by Equations (5) and (6) below:

(5) $$\rho_{o}=M/V_{m}=(P_{o}\cdot M)/(Z_{o}\cdot R_{u}\cdot T_{o}),$$

(6) $$c_{o}=\sqrt{Z_{o}\cdot \kappa_{o}\cdot \frac{R_{u}}{M}\cdot T_{o}},$$

where ρ is the gas density, g/m^3^; M is the molecular weight (molar mass), g/mol; $V_{m}$ is the molar volume, m^3^/mol; $R_{u}$ is the universal gas constant, set as 8.314 J/(mol⋅K); c is the sound velocity, m/s; and κ is the dimensionless isentropic index.

Considering Equations (5) and (6), based on the four measurable physical properties, the isentropic index under operating conditions can be further correlated and determined, as shown in Equation (7) below:

(7) $$\kappa_{o}=c_{o}^{2}\cdot \rho_{o}/P_{o},$$

For ideal gases, the isentropic index is typically 1.67 for monatomic gases, 1.40 for diatomic gases, and ranges from 1.1 to 1.3 for polyatomic gases. Under conventional physical conditions, the complexity of a gas molecule increases with the number of constituent atoms, resulting in to a smaller isentropic index. Therefore, the isentropic index can be used to characterize the molecular composition of a gas under specific conditions. Monoatomic gases behave as ideal gases at normal temperature and pressure because of negligible intermolecular forces and molecular volumes, leading to a compression factor close to 1. As molecular complexity increases, intermolecular interactions strengthen, and the compression factor of polyatomic gases deviates more significantly from unity than that of monatomic or diatomic gases. In summary, a positive correlation exists between the isentropic index and the compression factor: a lower isentropic index indicates a more complex molecular structure and a greater deviation of the compression factor from the ideal state, and vice versa.

**Figure 2:**
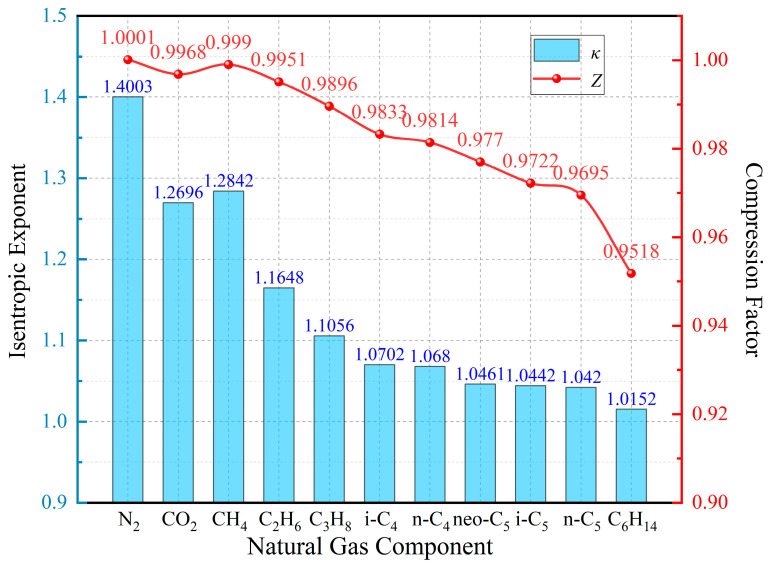
Schematic representation of the isentropic exponents and compression factors for common natural gas components under conditions of 101.325 kPa and 343 K.

Figure 2 presents the isentropic indices and compression factors for common natural gas components. To maintain heavy hydrocarbon gases in the gaseous state, environmental conditions are set at 101.325 kPa and 343 K. Comparison of the isentropic indices and compression factors under these conditions shows a clear positive correlation across typical natural gas components. This observation suggests that the compression factor can be reliably approximated by measuring the isentropic index.

#### 2.1.3. Correlation Analysis of Calorific Value Characteristics

Based on the temperature, pressure, sound velocity, density, and isentropic index of natural gas under various operating conditions, the compression factor can be estimated. This value, along with the measured density and Equation (5), enables for the calculation of the molecular weight, as shown in Equation (8):

(8) $$M=(\rho_{o}\cdot Z_{o}\cdot R_{u}\cdot T_{o})/P_{o},$$

**Figure 3:**
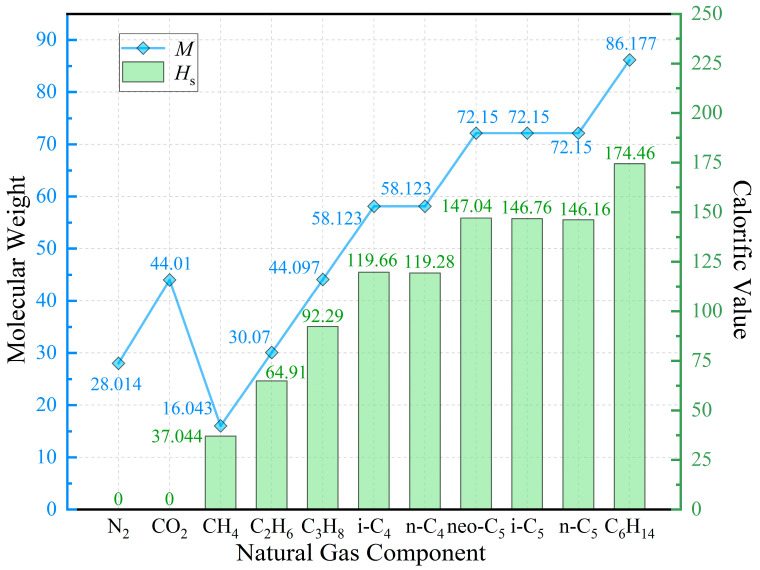
Schematic representation of molecular weights and calorific values of common natural gas components under standard conditions.

According to relevant standards [26], the molecular weights and calorific values of common natural gas components under standard conditions are depicted in Figure 3. From the figure, it is evident that the calorific value of natural gas originates primarily from alkane-type components (C_1_~C_6_), while non-alkane components (N_2_, CO_2_) contribute no calorific value.

The calorific values of the individual gases shown in Figure 3 are normalized by their molecular weights, as presented in Table 1 below. The data indicate that among the common natural gas components, the calorific values of alkane gases show minimal variation, while non-alkane gases have zero calorific values. Consequently, the overall calorific value of natural gas demonstrates an approximately linear correlation with the molecular weight of its alkane constituents.

The direct measurement of the molecular weight of alkane gases in natural gas presents technical challenges without a thorough understanding of its specific composition. However, the isentropic index provides a useful alternative: non-alkane gases typically exhibit significantly higher isentropic indices than alkane gases, making the isentropic index a key parameter for characterizing the alkane content. In summary, a reliable prediction model for natural gas properties can be constructed using the isentropic index in combination with molecular weight, thereby enabling accurate calorific value estimation.

#### 2.1.4. Hypothetical Calculation Example and Process Description

Based on the preceding analysis, it is possible to achieve real-time measurement of parameters such as temperature, pressure, density, sound velocity, and flow rate by leveraging the intrinsic correlations between various physical parameters of natural gas and utilizing industrial sensing instruments such as temperature/pressure transmitters, vibrating density meters, and ultrasonic flow meters. This allows for the calculation of core energy measurement parameters such as compression factor and calo-rific value, which completes the natural gas energy measurement process.

**Figure 4:**
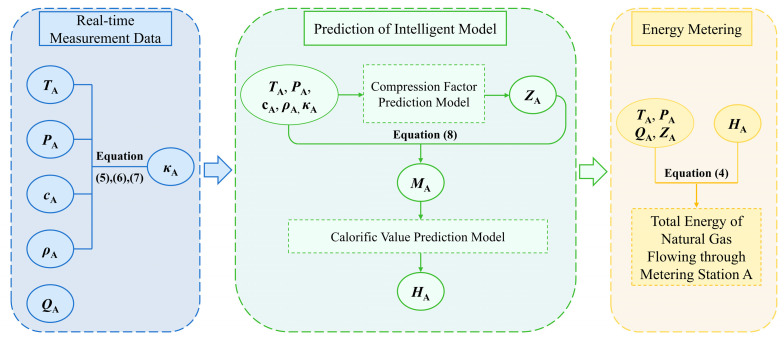
Flowchart of Hypothetical Example Calculation Process.

To explain the aforementioned implementation process, this paper presents a hypothetical calculation example using simplified numerical values, as shown in Figure 4. Assume that the temperature, pressure, flow rate, and composition of natural gas passing through metering station A remain constant over an hour. The detailed molar fraction information for each component of natural gas is as follows (unit: %mol/mol), Nitrogen(N_2_): 1.28, Carbon dioxide(CO_2_): 1.92, Methane(CH_4_): 95.56, Ethane(C_2_H_6_): 0.46, Propane(C_3_H_8_): 0.15, Butane(n-C_4_H_10_): 0.12, Isobutane(i-C_4_H_10_): 0.15, Pentane(n-C_5_H_12_): 0.12, Isopentane(i-C_5_H_12_): 0.13, Neopentane(neo-C_5_H_12_): 0.09, and Hextane(C_6_H_14_): 0.02. Physical property parameters measured by industrial sensor instruments are as follows: Temperature (*T*_A_): 293.15 K, Pressure (*P*_A_): 0.4 MPa, Flow rate (*Q*_A_): 10,000 m^3^/h, Density ($\rho_{A}$): 2.8395 kg/m^3^, Sound velocity (*c*_A_): 428.08 m/s. *T*_s_ and *P*_s_ denote the temperature and pressure under standard conditions, with default values of 293.15 K and 101.325 kPa.

The hypothetical calculation example follows this computational process:

(1) First, based on the real-time measured natural gas physical property data (*P*_A_, $\rho_{A}$, *c*_A_), combined with Equation (7) derived from the simultaneous solution of Equations (5) and (6), the isentropic exponent can be calculated ($\kappa_{A}=c_{A}^{2}\cdot \rho_{A}/P_{A}=$ 1.3008).

(2) Secondly, based on the interrelationships among various physical properties of natural gas, inputting relevant physical parameters (*T*_A_, *P*_A_, $\rho_{A}$, *c*_A_, $\kappa_{A}$) into the intelligent compression factor prediction model enables further prediction of the compression factor under operating conditions (*Z_A_* = 0.99217).

(3) Thirdly, based on the physical properties of natural gas (*T*_A_, *P*_A_, $\rho_{A}$, *Z*_A_), its molecular weight can be calculated using Equation (8) ($M_{A}=(\rho_{A}\cdot Z_{A}\cdot R_{u}\cdot T_{A})/P_{A}=17.16$ (g/mol)).

(4) Similarly, input the aforementioned natural gas physical property parameters (*T*_A_, *P*_A_, *c*_A_, $\rho_{A}$, $\kappa_{A}$, *M*_A_, *Z*_A_) into the intelligent calorific value prediction model to forecast its calorific value (*H*_A_ = 36.692 (MJ/m^3^)).

(5) Finally, based on five parameters—natural gas temperature (*T*_A_), pressure (*P*_A_), the compression factor (*Z*_A_), the calorific value (*H*_A_), and flow rate (*Q*_A_)—and using Equation (4), the energy measurement for natural gas at metering station A over one hour was performed, ultimately yielding its total energy as 1,459,918.7 (MJ). ($E_{A}=\frac{P_{A}\cdot T_{s}}{T_{A}\cdot P_{s}}\cdot \frac{H_{A}}{Z_{A}}\cdot t\cdot q=$1,459,918.7 (MJ)).

The correlation between the properties of natural gas in this paper refers to directional relationships, which are used to summarize the direction of change between parameters rather than linear correlations at the statistical level. The essence of the correlation presented in this paper is found in examining the trend-based relationship between the measured physical properties of natural gas and the key energy measurement parameters, specifically the compression factor and calorific value.

### 2.2. Sample Dataset Construction

Intelligent learning algorithms systematically analyze system behavior and evolutionary patterns from a macro perspective [30,31], requiring large datasets for effective model training. As described above, the prediction of compression factor and calorific value relies on datasets constructed from different dimensions:

(1) Compression factor prediction model: A five-dimensional dataset has been utilized. The input features include temperature, pressure, sound velocity, density, and isentropic index of natural gas under operating conditions, with the compression factor as the output label.

(2) Calorific value prediction model: This model is based on the five-dimensional dataset and includes the predicted compression factor and density to determine molecular weight. The resulting seven-dimensional dataset includes temperature, pressure, sound velocity, density, isentropic index, compression factor, and molecular weight as input features, with calorific value as the output label.

**Figure 5:**
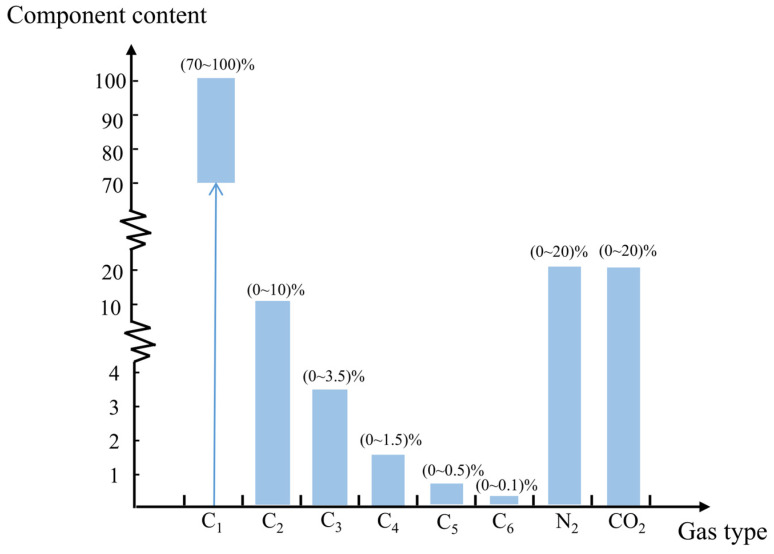
Schematic of artificially defined ranges for natural gas components (The arrow denotes the lower limit (70%) of the content range).

Due to the limited availability of natural gas samples with diverse compositions, the component compositions can be manually defined. For each composition, the compression factor and corresponding input-output pairs for calorific value prediction are systematically calculated under various operating conditions using standard methods. This study adheres to the common natural gas component ranges outlined in GB/T 17747.2-2011: Calculation of Natural Gas Compression Factors, Part 2: Calculation Using Molar Composition (Figure 5), generating a dataset covering 200 distinct compositions [14].

To analyze thermodynamic characteristics under diverse operating conditions, 50 operating conditions were systematically selected in this study, covering a temperature range of (253~333) K and a pressure range of (0~10) MPa (detailed in Table 2). The sample dataset combines 200 distinct natural gas compositions with these 50 operating conditions, resulting in a total of 10,000 data points, generated following standard calculation methods.

**Figure 6:**
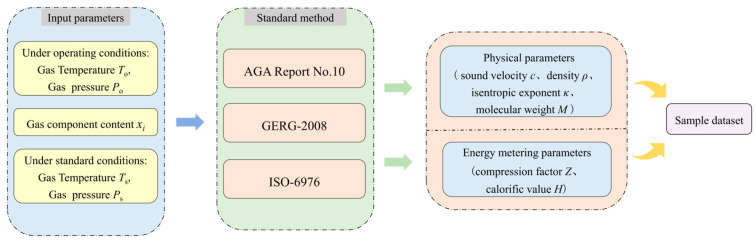
Flowchart of sample dataset construction [32,33,34,35].

Based on established research, AGA REPORT NO. 10 [32] provides the standard method for calculating the sound velocity of natural gas, while ISO-6976 [33] is widely used to determine the calorific value. The AGA8-92DC and GERG-2008 state equations are used to calculate the compression factor and density, with GERG-2008 providing superior accuracy [34,35]. Using AGA REPORT NO. 10, ISO-6976, and the GERG-2008 state equations, the input features and output labels of the compression factor and calorific value prediction models were systematically calculated for each natural gas composition under all operating conditions. The dataset construction process is illustrated in Figure 6. For subsequent intelligent learning-based prediction studies, the dataset is split into a training set (80%) and a testing set (20%).

Because input features vary in units and scales, features with larger numerical ranges can dominate during model training, potentially skewing feature importance and reducing model accuracy [36,37]. To address this, the dataset is normalized by linearly transforming each feature to the [0, 1] interval using Equation (9):

(9) $$x\prime =\frac{x-x_{\min}}{x_{\max}-x_{\min}},$$

where x′ is the normalized sample data; x is the original sample data before normalization; and $x_{\max}$ and $x_{\min}$ are the maximum and minimum values of the sample data, respectively.

## 3. Prediction Model Construction

**Figure 7:**
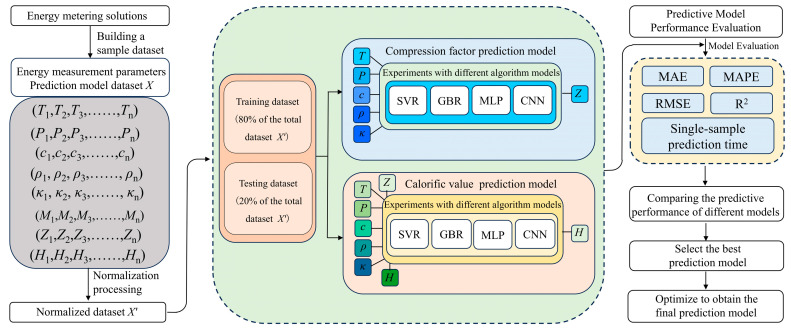
Flowchart of the construction process for a natural gas compression factor and calorific value prediction models (The arrows denote the direction of process progression.).

Figure 7 presents the flowchart for constructing a prediction model for the natural gas compression factor and calorific value. Using the previously constructed natural gas dataset, four models—Support Vector Regression (SVR), Gradient Boosting Regression (GBR), Multi-Layer Perceptron (MLP), and Convolutional Neural Network (CNN)—were trained and tested. Model performance was evaluated using five metrics: Mean Absolute Error (MAE), Root Mean Square Error (RMSE), Mean Absolute Percentage Error (MAPE), Coefficient of Determination (R^2^) and Single-sample prediction time. By comparing the evaluation metrics across these algorithms for both prediction tasks, the optimal models for compression factor and calorific value were selected, and the best-performing prediction model was determined through optimization.

### 3.1. Models and Evaluation Indicators

Given the characteristics of compression factor and calorific value prediction tasks, the chosen algorithms must satisfy the following criteria:

(1) Regression problem adaptability: The task is a supervised learning regression problem; the algorithm must effectively predict continuous variables.

(2) Feature-data compatibility: The algorithm should accurately capture the non-linear relationships between physical property data and target parameters to ensure reliable and stable predictions.

(3) Low model complexity: Overly complex models can be difficult to interpret and prone to overfitting. Simpler models are preferred, provided they achieve sufficient accuracy.

To meet these requirements, SVR, GBR, MLP, and CNN were employed to construct prediction models:

- SVR maps data to a high-dimensional space using a kernel function and identifies the optimal hyperplane [38].
- GBR iteratively fits weak learners and corrects residuals to approximate the true regression function [39].
- MLP is flexible regarding input data structure and can process data of arbitrary shape and dimension [40].
- CNN reduces the dimensionality of input data while extracting spatial features [41].

To quantitatively evaluate model performance, five metrics were used: RMSE, MAE, MAPE, and R^2^, defined in Equations (10)–(13), single-sample prediction time can serve as a key indicator for evaluating the prediction efficiency of a model.

(10) $$\mathrm{MAE}=\frac{100\%}{n}\sum_{i=1}^{m}\left|{\hat{y}}_{i}-y_{i}\right|,$$

(11) $$\mathrm{MAPE}=\frac{100\%}{n}\sum_{i=1}^{m}\left|\frac{{\hat{y}}_{i}-y_{i}}{y_{i}}\right|,$$

(12) $$\mathrm{RMSE}=\sqrt{\frac{1}{m}\cdot \sum_{i=1}^{m}{\left({\hat{y}}_{i}-y_{i}\right)}^{2}},$$

(13) $$R^{2}=1-\frac{\sum_{i=1}^{m}{\left({\hat{y}}_{i}-y_{i}\right)}^{2}}{\sum_{i=1}^{m}{\left({\bar{y}}_{i}-y_{i}\right)}^{2}},$$

where m denotes the sample size; $y_{i}$ is the true sample value; ${\hat{y}}_{i}$ is the predicted value from the machine learning model; and ${\bar{y}}_{i}$ is the mean of the true sample values.

Among these indicators, smaller MAE, MAPE, and RMSE values indicate more accurate model predictions, shorter single-sample prediction time indicates higher predictive efficiency of the model, while an R^2^ value closer to 1 signals a better fit of the regression model.

### 3.2. Model Training

#### 3.2.1. Compression Factor Prediction Model

The previously constructed sample dataset was used for both training and testing the models. To ensure that each model achieved its optimal predictive performance, hyperparameters were tuned using grid search methods [42,43]. Table 3 summarizes the hyperparameter configurations at which each model exhibited its best predictive capability.

**Figure 8:**
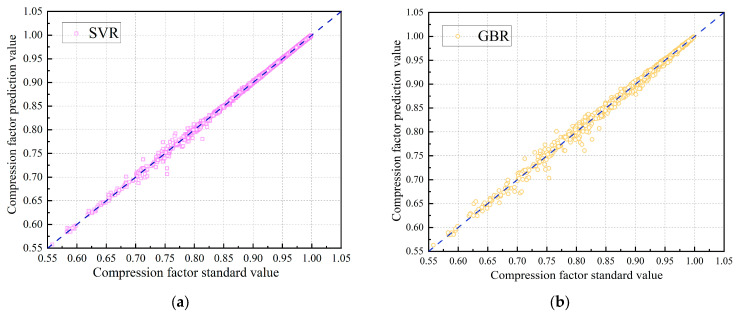

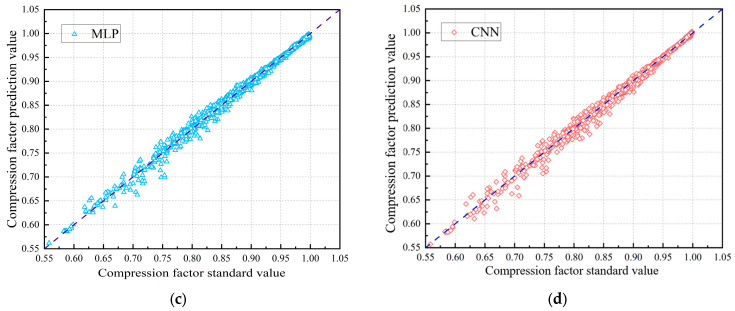
Comparison of predicted and standard compression factor values for each model. (**a**) SVR Model; (**b**) GBR Model; (**c**) MLP Model; (**d**) CNN Model.

In addition, Figure 8 presents a scatter plot comparing predicted compression factor values from each model against the ideal perfect prediction line superimposed as a reference. The dispersion of points around the reference line provides a visual assessment of prediction accuracy: smaller deviations indicate more accurate predictions.

As illustrated in Figure 8, all four models accurately and efficiently predict natural gas compression factors. Performance metrics in Table 4 indicate that the SVR model outperforms the others. Its superior performance can be attributed to the kernel function, which effectively captures non-linear relationships even with limited input features, and its robustness to outliers ensures stable and reliable predictions.

#### 3.2.2. Calorific Value Prediction Model

**Figure 9:**
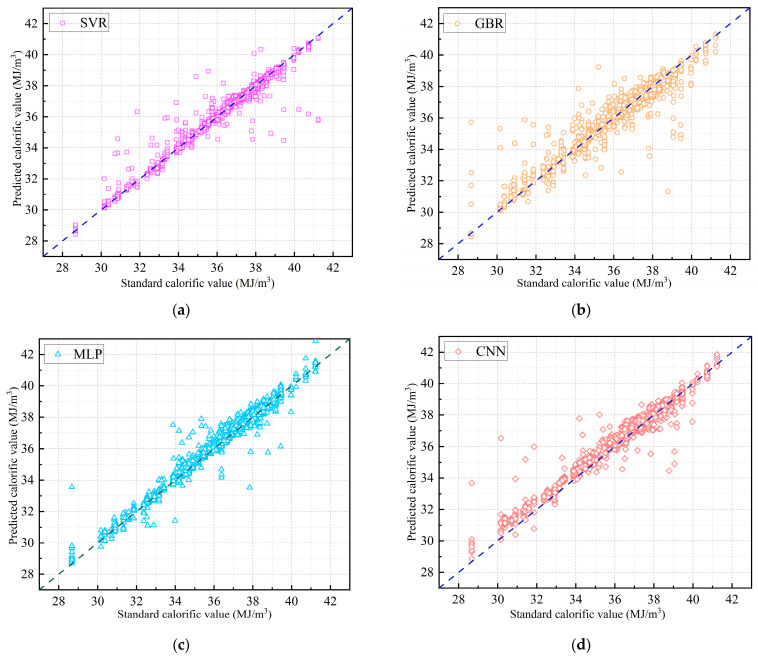
Comparison of predicted and standard calorific values for each model. (**a**) SVR Model; (**b**) GBR Model; (**c**) MLP Model; (**d**) CNN Model.

Four algorithmic models were used to predict the calorific value of natural gas. Table 5 summarizes the hyperparameter configurations for each model when optimal prediction performance was achieved. Figure 9 displays a scatter plot of predicted versus actual calorific values for each model. The dispersion of points around the ideal prediction line provides a visual evaluation of model accuracy.

Performance metrics in Table 6 indicate that SVR and MLP achieved the best results. Specifically, MLP exhibits the lowest MAE and MAPE, indicating minimal relative deviation between predicted and standard values. Additionally, SVR achieves superior RMSE and R^2^, reflecting stronger capability in capturing data trends and fitting quality. Meanwhile, the MLP model demonstrates the fastest single-sample inference speed; thus, it can be prioritized as the preferred prediction model for large-scale sample forecasting tasks.

### 3.3. Model Switching Method Based on Feature Range

**Figure 10:**
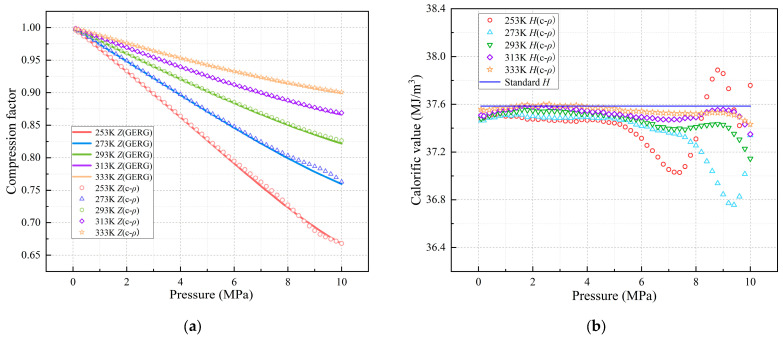
Comparison of compression factor and calorific value predictions versus standard values for natural gas under varying operating conditions. (**a**) Compression Factor Prediction; (**b**) Calorific Value Prediction (c-ρ: Predictions Based on Density-Sound Velocity Correlation).

Overall, outliers frequently occur when intelligent learning algorithms process natural gas sample datasets for training and prediction. To investigate the causes of outliers, a separate set of natural gas samples from a certain region in China (excluded from the training dataset) was analyzed. As shown in Figure 10, the relative error of predicted values increases significantly under medium- to high-pressure conditions; notably, low-temperature environments can further exacerbate this error amplification trend. In these conditions, natural gas approaches a supercritical state, exhibiting properties of both gas and liquid, which leads to prediction failure and elevated errors.

**Figure 11:**
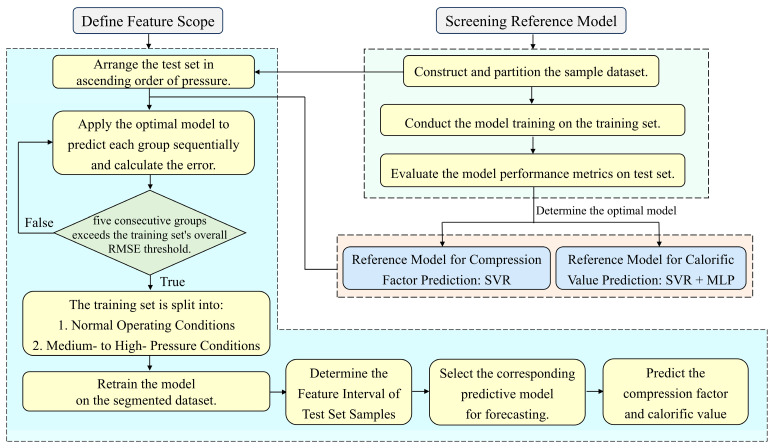
Flowchart of the model switching method based on feature range.

Inspired by the core tenets of adaptive algorithms and the Mixture of Experts (MoE) algorithm [44,45], a model switching method based on feature range is proposed to address this issue. The overall principle of MSM-FR is as follows (Figure 11):

(1) The natural gas sample dataset was randomly split into a training set (80%) and a testing set (20%). Based on this, a multi-model reference prediction framework was developed, which included SVR, GBR, MLP, and CNN. The compression factor and calorific value prediction models were trained with measured physical properties as input. Five metrics were used to evaluate the overall performance of the model: MAE, RMSE, MAPE, R^2^, and single-sample prediction time.

(2) According to the evaluation results, the SVR model outperformed all four metrics and was chosen as the baseline model for the compression factor prediction task. In the calorific value prediction task, the SVR model demonstrated superior performance in RMSE and R^2^, while the MLP model showed greater advantages in MAE and MAPE. The choice between the two models should be based on the specific operational requirements.

(3) Under medium- to high-pressure pipeline transmission conditions, natural gas has properties similar to two-phase gas-liquid behavior, reducing the predictive accuracy of a single model. As a result, after completing the reference model evaluation, differentiated feature interval partitioning schemes must be developed for various prediction tasks, and models must be trained separately for each partitioned sub-interval.

The actual workflow is as follows: In the compression factor prediction task, arrange the measured physical property features of the test set in ascending order of pressure. Then, predict and calculate the error for each group sequentially. When the prediction error for five consecutive groups exceeds the training set’s overall RMSE threshold, extract the density and sound velocity values from the first group of five samples. These values serve as the foundation for distinguishing between conventional operating conditions and medium to high-pressure conditions. For the caloric value prediction task, the compression factor, which is already an input feature, is selected directly as the interval partitioning parameter. Additionally, given the SVR model’s superior generalization capability and robustness in the calorific value prediction task, the benchmark model for this prediction task was also selected as SVR prior to implementing feature subinterval partitioning. Table 7 shows the feature range classification and model adaptation table.

The primary reason for selecting these parameters is that natural gas with different compositions exhibits significant property variations under the same temperature and pressure conditions. Density, sound velocity, and compression factor all describe the combined effects of temperature and pressure on natural gas properties. Compared to single-pressure parameter partitioning methods, this approach significantly improves interval segmentation accuracy.

(4) Once the feature subinterval partitioning is complete, independent prediction models must be trained for each interval. To perform compression factor predictions, separate SVR models are constructed for conventional operating conditions and medium- to high-pressure conditions subintervals. The compression factor prediction model trained for the conventional operating condition is denoted as SVR-Z_1_, whereas the counterpart tailored for the medium- to high-pressure regime is designated as SVR-Z_2_. Model selection for the calorific value prediction task is differentiated by the following characteristics: The SVR model is well-suited for the medium- to high-pressure operating condition interval, thanks to its advantages in RMSE and R^2^ metrics, as well as its strong robustness. The calorific value prediction model trained for this scenario is denoted as SVR-H. The MLP model, which outperforms the MAE and MAPE metrics, is better suited to the high-precision prediction requirements of the conventional operating condition interval, and the corresponding prediction model is denoted as MLP-H.

(5) Finally, determine the feature interval to which the test set samples belong, and then apply the optimal model trained for that interval to accurately predict the compression factor and calorific value.

The baseline model was trained on the training set and systematically validated via experiments using the test set. Through this procedure, the demarcation criteria for conventional and medium- to high-pressure operating conditions were established for both the compression factor and calorific value prediction tasks, alongside tailored model switching strategies for each task. Detailed specifications are elaborated below and summarized in Table 7.

For the compression factor prediction task:

(1) When the density feature value of the input sample is less than or equal to 52.26 g/m^3^ or the sound velocity feature value is greater than or equal to 400.54 m/s, the sample is classified as operating under conventional conditions, with the SVR-Z_1_ model employed for compression factor prediction.

(2) When the density feature value of the input sample exceeds 52.26 g/m^3^ and the sound velocity feature value is below 400.54 m/s, the sample is classified as operating under medium- to high-pressure conditions, with the SVR-Z_2_ model employed for compression factor prediction.

For the calorific value prediction task:

(1) When the compression factor feature value of the input sample is greater than or equal to 0.8542, the sample is classified as operating under conventional conditions, with the MLP-H model employed for calorific value prediction.

(2) When the compression factor feature value of the input sample falls below 0.8542, the sample is classified as operating under medium -to high-pressure conditions, with the SVR-H model employed for calorific value prediction.

**Figure 12:**
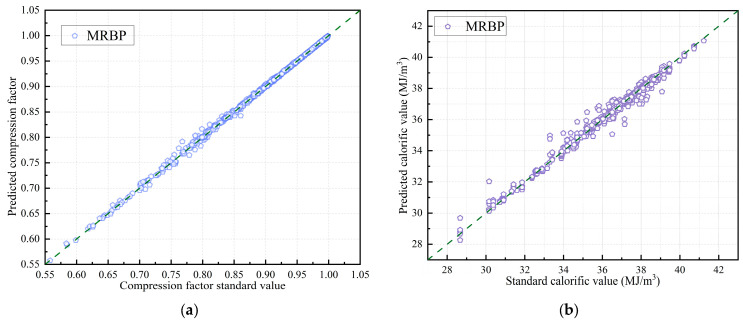
Comparison of MSM-FR model predictions with standard values: (**a**) Compression Factor Prediction; (**b**) Calorific Value Prediction.

**Figure 13:**
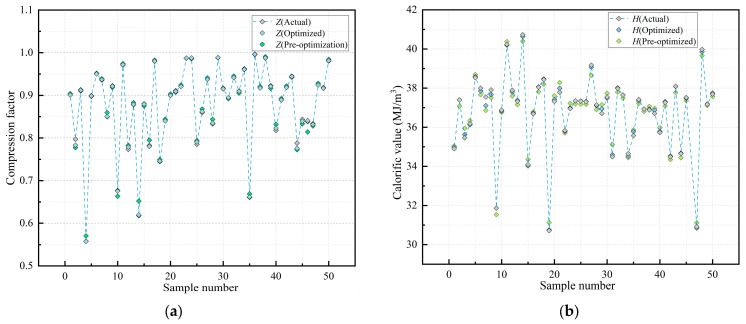
Comparison of model predictions before and after optimization: (**a**) Compression Factor; (**b**) Calorific Value.

The MSM-FR optimization algorithm was applied to train and predict the sample dataset. The evaluation of model performance involved comparing predicted values against standard values, as shown in Figure 12. Additionally, Figure 13 further illustrates the improvement achieved by MSM-FR, comparing predicted values before and after optimization. It is evident that, relative to the optimization models, the MSM-FR-optimized model produces predictions for both compression factor and calorific value that are closer to the standard values.

Table 8 summarizes the performance metrics of the MSM-FR optimization algorithm. The results demonstrate a clear improvement in prediction accuracy for both the com-pression factor and calorific value, and although the proposed MSM-FR optimization algorithm leads to a slight increase in single-sample prediction time compared with the optimal single model, it achieves a favorable trade-off between efficiency and accuracy. In the compression factor prediction model, the MAE, RMSE, and MAPE decreased from 0.00267, 0.0052, and 0.32 to 0.00118, 0.0030, and 0.14, respectively. At the same time, the R^2^ increased from 0.9963 to 0.9987, indicating an improved goodness-of-fit between the predicted and standard values. For the calorific value prediction model, the MAE, RMSE, and MAPE decreased from 0.2481, 0.415, and 0.69 to 0.1583, 0.331, and 0.44, respectively. On the other hand, R^2^ increased from 0.9641 to 0.9736, reflecting a significant reduction in prediction errors and improved model reliability. Overall, the MSM-FR optimization algorithm effectively enhances the accuracy and robustness of both compression factor and calorific value predictions.

## 4. Case Study

### 4.1. Actual Flow Test

**Figure 14:**
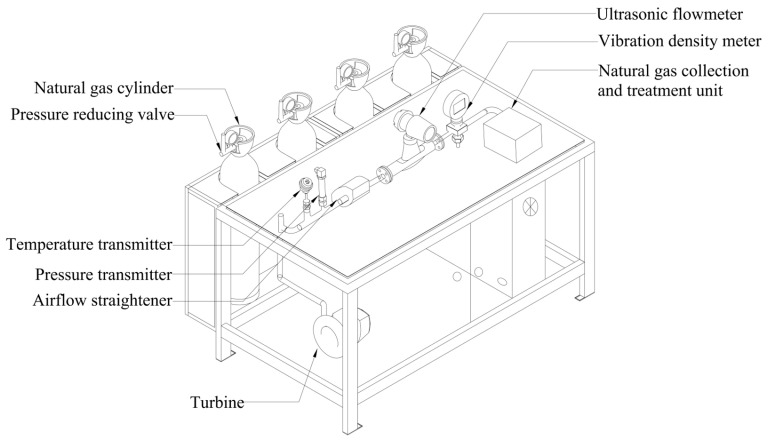
Structural diagram of the natural gas energy metering test bench.

To validate the method under real-flow conditions, a natural gas energy metering test device was established. The structural diagram of the device is shown in Figure 14. The device includes an airflow straightener to enhance the measurement accuracy of the ultrasonic flow meter, in addition to a turbine and natural gas collection/treatment unit to purge and safely collect natural gas from the pipeline.

Four types of natural gas cylinders were used as the gas source, with their component compositions detailed in Table 9. During testing, each cylinder was opened, and the pressure was controlled by adjusting a pressure-reducing valve. Every pressure point was tested three times. Sound velocity and density were recorded and averaged to serve as input features for the intelligent learning algorithm. These inputs were then used to predict the compression factor and calorific value of natural gas under varying temperature and pressure conditions.

### 4.2. Experimental Results

**Figure 15:**
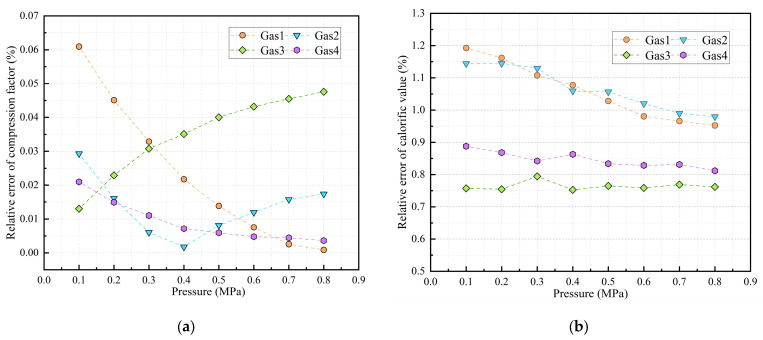
Relative errors in predicted compression factor and calorific value of natural gas under actual flow conditions. (**a**) Compression Factor Prediction; (**b**) Calorific Value Prediction.

The MSM-FR optimization algorithm was applied to predict the compression factor and calorific value of four natural gas samples under multiple operating conditions, using physical property parameters collected in real time by the test device. The predicted values were systematically compared with standard values calculated according to industry norms to assess prediction accuracy and model reliability. The results are presented in Figure 15.

Furthermore, Table 10 summarizes the maximum absolute error (*AE*_max_) and maxi-mum relative error (*RE*_max_) of the compression factor and calorific value of each gas sample group. The data indicate that, under actual flow conditions, the maximum absolute error of the compression factor predictions across the four gas groups was 0.00061, with a maximum relative error of 0.061%. This demonstrates minimal prediction error and strong model performance. The maximum absolute error for calorific value predictions was 0.478 MJ/m^3^, with a maximum relative error of 1.19%. The relative errors of total energy for the four gas groups were 1.21%, 1.17%, 0.80%, and 0.95%, respectively. Notably, gas samples 1 and 2 exhibited relatively larger errors, which can be attributed to their higher calorific values and a limited number of concentrated data points, resulting in reduced model generalization capability.

## 5. Conclusions

This paper proposes a natural gas energy metering method based on the density-sound velocity correlation, enabling accurate metering without the need for a gas chromatograph. The study involved constructing a multidimensional dataset of 10,000 sample sets, integrating multiple intelligent learning algorithms to establish predictive models for compression factor and calorific value, proposing the MSM-FR optimization algorithm to enhance prediction accuracy, and performing real-flow experiments to evaluate practical applicability. The main findings of this study are summarized as follows:

(1) This study proposes measuring key physical parameters, including temperature, pressure, sound velocity, density, isentropic index, and molecular weight, and integrating them with intelligent learning algorithms, thereby enabling accurate real-time energy metering of natural gas with the measurement frequency drastically improved to the second level.

(2) In accordance with natural gas standards, this study constructed a comprehensive sample dataset for the comparative experiments of different intelligent learning algorithms. In predicting the compression factor, the Support Vector Regression (SVR) model demonstrated superior performance, while for predicting calorific value, both the SVR and Multi-layer Perceptron (MLP) models showed distinct advantages.

(3) Based on the results of the model analysis, the MSM-FR optimization algorithm was proposed to further enhance prediction capability. The performance metrics for the compression factor prediction model are as follows: MAE = 0.00118, RMSE = 0.0030, MAPE = 0.14%, and R^2^ = 0.9987. For the calorific value prediction model, the corresponding metrics are: MAE = 0.1583, RMSE = 0.331, MAPE = 0.44%, and R^2^ = 0.9736, indicating high predictive accuracy and robustness.

(4) A natural gas energy metering real-flow test rig was constructed, and the predictive models of the compression factor and calorific value were applied to real-flow conditions. Results demonstrated that the proposed method is very effective for natural gas energy metering. In the tests, the maximum prediction errors for the compression factor and calorific value were 0.061% and 1.19%, respectively, with the maximum relative error for total energy at 1.21%, confirming the reliability and accuracy of the method.

(5) The natural gas sample data used in this paper consist of 11 common components, including methane, ethane, nitrogen, carbon dioxide, and others. However, in practical applications, natural gas may contain uncommon components such as hydrogen, hydrogen sulfide, and argon. Due to differences in the physical properties of various gases, it is necessary to further enrich the sample dataset to train the prediction model and evaluate the error generated by this method when dealing with natural gas composed of additional components. In addition, follow-up research can be conducted based on the physical properties selected in this paper, incorporating other natural gas physical properties as feature values to further reduce the error of the physical property correlation method in natural gas energy metering.

## Figures and Tables

**Table 1:** Calorific value-to-molecular weight ratios of common natural gas components under standard conditions.

	N_2_	CO_2_	C_1_	C_2_	C_3_	i-C_4_	n-C_4_	neo-C_5_	i-C_5_	n-C_5_	C_6_
*H*_s_/*M*	0	0	2.31	2.16	2.09	2.06	2.05	2.04	2.03	2.03	2.02

**Table 2:** Operating conditions for dataset construction.

Temperature (K)	Pressure (MPa)									
253	0.1	0.2	0.4	0.8	1.6	2.5	4.0	6.0	8.0	10.0
273	0.1	0.2	0.4	0.8	1.6	2.5	4.0	6.0	8.0	10.0
293	0.1	0.2	0.4	0.8	1.6	2.5	4.0	6.0	8.0	10.0
313	0.1	0.2	0.4	0.8	1.6	2.5	4.0	6.0	8.0	10.0
333	0.1	0.2	0.4	0.8	1.6	2.5	4.0	6.0	8.0	10.0

**Table 3:** Hyperparameter configurations for compression factor prediction models.

	SVR	GBR	MLP	CNN
Basic Structural Parameters	Kernel: ‘rbf’ Gamma: ‘auto’	N_estimators: 235; Max_depth: 5; Min_samples_split: 12; Max_features: 4;	Hidden layer sizes: (64, 32, 16); Batch_size: 64; Epochs: 50;	Fully connected layer sizes: (64, 32, 16) Conv layer: [Kernel size: (1,3), Padding: (0,1)]; Max pooling layer: [Kernel size: (1,2), Stride: (1,2)]; Batch_size: 64; Epochs: 102;
Training-related parameters	C = 10 Epsilon: 0.01 Degree: 5	Learning rate: 0.01; Loss: ‘Least Squares’	Learning rate: 0.025; Activation: ‘Tansig’; Optimizer: ‘adam’; Loss: ‘mseloss’; Dropout: 0.2;	Learning rate: 0.007; Activation: ‘Relu’; Optimizer: ‘adam’; Loss: ‘mseloss’; Dropout: 0.2;

**Table 4:** Performance indicators for compression factor prediction models.

Model	MAE	RMSE	MAPE (%)	R^2^	Single-Sample Prediction Time (ms)
SVR	0.00267	0.0052	0.32	0.9963	0.2374
GBR	0.00327	0.0061	0.39	0.9948	0.4080
MLP	0.00417	0.0072	0.50	0.9929	0.3552
CNN	0.00498	0.0083	0.60	0.9906	0.7584

**Table 5:** Hyperparameter configurations for calorific value prediction models.

	SVR	GBR	MLP	CNN
Basic Structural Parameters	Kernel: ‘rbf’ Gamma: ‘scale’	N_estimators: 250; Max_depth: 10; Min_samples_split: 3; Max_features: 4;	Hidden layer sizes: (256, 128, 64, 16); Batch_size: 64; Epochs: 50;	Fully connected layer sizes: (896, 256, 128, 64, 16) Conv layer: [Kernel size: (1,3), Padding: (0,1)]; Batch_size: 64; Epochs: 550;
Training-related parameters	C = 20 Epsilon: 0.06 Degree: 5	Learning rate: 0.04; Loss: ‘Least Squares’	Learning rate: 0.001; Activation: ‘Tansig’; Optimizer: ‘adam’; Loss: ‘mseloss’; Dropout: 0.2;	Learning rate: 0.005; Activation: ‘Relu’; Optimizer: ‘adam’; Loss: ‘mseloss’; Dropout: 0.2;

**Table 6:** Performance indicators for calorific value prediction models.

Model	MAE	RMSE	MAPE (%)	R^2^	Single-Sample Prediction Time (ms)
SVR	0.2741	0.385	0.76	0.9641	0.5257
GBR	0.2840	0.570	0.79	0.9222	0.4857
MLP	0.2481	0.415	0.69	0.9577	0.4394
CNN	0.2925	0.481	0.81	0.9448	0.7580

**Table 7:** Feature range classification and model adaptation table.

Prediction Target	Feature Range	Algorithm Model
Compression factor	$\rho \leq 52.26\;(\mathrm{kg}/m^{3})\|c\geq 400.54\;(m/s)$	SVR-Z_1_
	$\rho >52.26\;(\mathrm{kg}/m^{3})\;\&\;c<400.54\;(m/s)$	SVR-Z_2_
Calorific value	Z≥0.8542	MLP-H
	Z<0.8542	SVR-H

**Table 8:** Performance metrics of the MSM-FR optimization algorithm.

Prediction Target	Model	MAE	RMSE	MAPE(%)	R^2^	Single-Sample Prediction Time (ms)
Compression factor	SVR-1	0.00054	0.0010	0.06	0.9995	0.2192
	SVR-2	0.00431	0.0070	0.57	0.9915	0.2234
	SVR	0.00118	0.0030	0.14	0.9987	0.2423
Calorific value	SVR	0.4858	0.716	1.32	0.8524	0.5274
	MLP	0.0840	0.133	0.24	0.9958	0.4419
	MLP + SVR	0.1583	0.331	0.44	0.9736	0.4653

**Table 9:** Detailed component composition of natural gas cylinders.

	$x_{N_{2}}$	$x_{CO_{2}}$	$x_{C_{1}}$	$x_{C_{2}}$	$x_{C_{3}}$	$x_{{\mathrm{n-C}}_{4}}$	$x_{{\mathrm{i-C}}_{4}}$	$x_{{\mathrm{n-C}}_{5}}$	$x_{{\mathrm{i-C}}_{5}}$	$x_{C_{6}}$
Gas1	2.99	0	85.1	8.92	2.99	0	0	0	0	0
Gas2	2.00	0	90.07	5.94	1.99	0	0	0	0	0
Gas3	2.02	2.52	94.135	0.521	0.201	0.1	0.1	0.15	0.152	0.101
Gas4	0.996	0.099	97.0883	1.01	0.201	0.101	0.101	0.153	0.1507	0.1

**Table 10:** Maximum absolute and relative errors of compression factor and calorific value for each natural gas sample group.

	Compression Factor		Calorific Value (MJ/m^3^)		Energy (MJ)
	*AE*_max_	*RE*_max_	*AE*_max_	*RE*_max_	*RE*_max_
Gas1	$2.1\cdot 10^{-4}$	0.021%	0.478	1.19%	1.21%
Gas2	$4.7\cdot 10^{-4}$	0.047%	0.447	1.14%	1.17%
Gas3	$1.7\cdot 10^{-4}$	0.017%	0.296	0.79%	0.80%
Gas4	$6.1\cdot 10^{-4}$	0.061%	0.331	0.88%	0.95%

## Data Availability

The raw data supporting the conclusions of this article will be made available by the authors upon request.

## Abbreviations

*E*	Total energy of natural gas
*t*_0_	Initial moment of energy metering
*t_n_*	Final moment of energy metering
*e*_s_(*t*)	Instantaneous energy flow as a function of time
*q*_s_(*t*)	Volume flow rate as a function of time
*H*_s_(*t*)	Calorific value as a function of time
*q*	Volume flow rate
*f*	Volume flow correction parameter
*P*	Pressure
*T*	Thermodynamic temperature
*Z*	Compression factor
$H_{s}^{o}$	Ideal calorific value of natural gas under standard conditions
$H_{s}$	Calorific value of natural gas under standard conditions
$H_{s,i}^{o}$	Ideal calorific value of each component in natural gas under standard conditions
*x_i_*	Molar fraction of each component
*n*	Total number of components in natural gas
*m*	Sample size
ρ	Gas density
*M*	Molecular weight
*V_m_*	Mole volume
*R_u_*	Universal gas constant
*c*	Sound velocity
κ	Isentropic index
*x*’	Normalised sample data
*x*	Original sample data before normalisation
*x*_max_	Maximum value of the sample data
*x_min_*	Minimum value of the sample data
*y_i_*	True sample value
${\hat{y}}_{i}$	Predicted value from the machine learning model
${\bar{y}}_{i}$	Mean of the true sample values
MAE	Mean absolute error
RMSE	Root mean square error
MAPE	Mean absolute percentage error
*R^2^*	Coefficient of determination
subscript	
*s*	Standard reference conditions
*o*	Operating conditions
